# Xiao-Shen-Formula, a Traditional Chinese Medicine, Improves Glomerular Hyper-Filtration in Diabetic Nephropathy via Inhibiting Arginase Activation and Heparanase Expression

**DOI:** 10.3389/fphys.2018.01195

**Published:** 2018-09-26

**Authors:** Xiaofei An, Maoxiang Zhang, Sisi Zhou, Tian Lu, Yongjun Chen, Lin Yao

**Affiliations:** ^1^School of Pharmaceutical Sciences, Guangzhou University of Chinese Medicine, Guangzhou, China; ^2^Department of Endocrinology, Jiangsu Province Hospital of Chinese Medicine, Affiliated Hospital of Nanjing University of Chinese Medicine, Nanjing, China; ^3^South China Research Center for Acupuncture and Moxibustion, Guangzhou University of Chinese Medicine, Guangzhou, China; ^4^Medical College of Acu-Moxi and Rehabilitation, Guangzhou University of Chinese Medicine, Guangzhou, China

**Keywords:** diabetic nephropathy, glomerular hyper-filtration, Xiao-Shen-Formula, arginase, nitric oxide, heparanase

## Abstract

Hyperglycemia induces glomerular hyper-filtration, which contributes to the development of diabetic nephropathy (DN), a condition that remains a challenge for treatment. The present study investigated the effect of Xiao-Shen-Formula (XSF) used for treatment of renal injury in type 1 DN mice model induced by streptozotocin (STZ) and its underlying mechanism in cultured human glomerular endothelial cell (hGECs). Studies were performed using control, diabetic DN, DN treated with XSF groups (1 g/kg/d, LXSF or 3 g/kg/d, HXSF) for 6 weeks and hGECs were post-treated with mice serum containing HXSF (MS-HXSF) and arginase inhibitor (ABH, 100 μM) in high glucose medium. HXSF treatment restored STZ-induced renal hyper-filtration, glomerulosclerosis, renal microvascular remodeling and the increased levels of systemic reactive oxidative species and inflammatory cytokines, accompanied by preventing the decreased expression of glomerular heparin sulfate and the increased levels of cortical heparanase and argianse2 protein and arginase activity. In hGECs study, MS-HXSF ameliorated the enhancement in arginase activity, the protein/mRNA expression of heparanase, mRNA levels of vascular cell adhesion molecule-1, intercellular adhesion molecule-1, monocyte chemoattractant protein-1 and permeability of hGECs monolayers as well as the depression of nitric oxide production. Besides all these protective effects, XSF blunted the mRNA expression of TNF-α *in vivo* and *vitro* studies as well, which was not changed by the post-treatment of ABH or HXSF plus ABH. This study demonstrated that the protective effect of XSF might be related with vascular prevention, anti-inflammation and anti-oxidation through intervening multi-targets including glomerular endothelial arginase-heparanase signaling pathway in DN model.

## Introduction

Diabetic nephropathy (DN) is one of the most common microvascular complications due to diabetes mellitus, however there is still a challenge for treatment (Fineberg et al., 2013). Proteinuria is not only the main clinical symptom and diagnostic indicator of DN, but also the primary cause of persistent renal damage (Jefferson et al., 2008; Coca et al., 2012; Vejakama et al., 2012). Current clinical treatment for DN proteinuria includes the control of blood glucose, blood pressure and the use of angiotensin-converting enzyme inhibitor or angiotensin receptor blocker and other drugs (UK Prospective Diabetes Study [UKPDS] Group, 1998a,b; Fowler, 2008; Vejakama et al., 2012). Clinical studies found that the degree of proteinuria was still progressively worsened, although patients with DN had accepted the above comprehensive treatment (Satirapoj and Adler, 2014). Therefore, it is important to identify the mechanisms involved in the development of proteinuria in the early stage of DN pathogenesis and look for new interventions in the prevention of DN.

Heparanase is known mammalian endoglycosidase that cleaves heparin sulfate (HS) (Wijnhoven et al., 2006) and finally results in the progression of proteinuria and renal failure (van den Hoven et al., 2007; Garsen et al., 2016a). The latest research has shown that heparanase is regulated by endothelial nitric oxide synthase (eNOS) -mediated nitric oxide (NO) production in glomerular endothelium (Garsen et al., 2016a). Arginase is another enzyme, which can reciprocally regulate production of NO by NOS through competition for their common substrate, L-arginine (Romero et al., 2008; Peyton et al., 2009). Accumulated studies raised the possibility that increased renal arginase activity and arginase 2 expression play a key role in pathogenesis of DN (Morris et al., 2011; Romero et al., 2013).

Xiao-Shen-Formula (XSF) is a widely used for the treatment of DN. Our previous studies have shown that XSF can significantly decrease the level of proteinuria, improve glomerular hyper-filtration and delay the degeneration of renal function in DN patients (Teng and An, 2016; An et al., 2017). However, the mechanisms of XSF in the treatment of DN have never been explored. The present study aims to investigate whether the ameliorative effect of XSF on diabetes-induced renal dysfunction associated with arginase and heparanase signaling.

## Materials and Methods

### Animals

Ten week-old male C57BL/6J mice were used in this study, which were obtained from the Animal Center of Guangzhou University of Chinese Medicine (GZUCM).

### Composition of Xiao-Shen-Formula

Xiao-Shen-Formula granules was provided by Jiangsu Tianjiang Pharmaceutical, Co., Ltd. (Jiangyin, China), which consists of *tuckahoe* (Fuling) (16.7%), *radix astragali* (Huangqi) (16.7%), *red paeonia* (Chishao) (11.1%), *luffa* (Sigualuo) (8.3%), *honeysuckle flower* (Yinhua) (11.1%), *radix curcumae* (Yujin) (8.3%), *cogongrass rhizome* (Baimaogen) (16.7%), and *radix achyranthis bidentatae* (Huainiuxi) (11.1%).

### Experiment Protocols

Four groups of mice were prepared: Control group, Diabetic nephropathy group (DN), DN treated with 1 g/kg/d XSF (LXSF), DN treated with 3 g/kg/d XSF (HXSF). For diabetic groups, mice received intraperitoneal (i.p.) injections of streptozotocin (STZ, 65 mg/kg) every other day for up to three injections. In the non-diabetic groups, citrate buffer (pH = 4.5), the vehicle of STZ, was injected in the same manner as in the diabetic groups. Mice with blood glucose levels > 350 mg/dL were considered diabetic. After 12 weeks diabetes, the animals in XSF treatment groups received XSF by gavage daily at a dose as indicated for another 6 weeks. Body weight and blood/urine glucose levels of each mouse were measured at the time of injections and 18 weeks after treatment. The dosage of XSF used in the experiments was calculated based on that for human, with 1 g/kg being equivalent to 3 g crude drugs/kg used in clinic. Blood glucose was measured in tail vein blood and serum and urinary creatinine levels were measured by the enzymatic colorimetric method. Every 2 weeks after drug administration, individual 24-h urine sample collections were performed using metabolic cages. Urinary albumin concentration was measured by Exocell kits using anti-mouse albumin antibody. Data were normalized to the urinary creatinine levels and expressed as the urinary microalbuminuria and creatinine ratio (mAlb/Cr). The creatinine clearance (CCr) was calculated and expressed as ml/min/100 g body weight. Kidney weight was measured right after animals were sacrificed. Fresh kidney cortical tissues were excised and stored at -80°C until further analysis.

### Preparation of XSF Containing Serum

Male C57BL/6J mice were administered by gavage with the adjuvant of XSF formula or HXSF (3 g/kg/d) once a day for 5 days, 2 h after the final dose, the mice were sacrificed and blood was collected. The blood samples were centrifuged at 2500 rpm for 15 min at 4°C, the serum was collected and incubated in a water both at 56°C for 30 min for inactivation, and stored at -80°C before use (Sun et al., 2017).

### Human Glomerular Endothelial Cell Culture

Human glomerular endothelial cells (Lonza, Walkersville, MD, United States) were grown in complete CSC medium, and maintained at 37°C in humidified 5% CO_2_ incubator. Cells were used between passages four and six for the experiments. Treatment of cells with normal (5.5 mM, HG) or high D-glucose-supplemented medium (25 mM, HG) was performed in basic CSC medium for 24 h or 14 days. As control for the osmotic effect of high D-glucose, L-glucose was added to the basic endothelial medium. Post-treatment of HGECs with arginase inhibitor ABH (100 μM, Corridor Pharmaceuticals, Baltimore, MD, United States), 20% control mouse serum [basal srum (BS)] or 20% HXSF mouse serum for 1 h after the HG treatment.

### Enzyme-Linked Immunosorbent Assay

The serum angiotensin II and aldosterone were measured by commercial ELISA kits (R&D, Wiesbaden, Germany) according to the operating instructions.

### PAS Staining

Half of the mouse kidney was fixed in 10% formalin buffer and then embedded in paraffin for light microscopic observation. Three sections of 5 μm thickness (an interval of 100 μm) for every animal were chosen using an unbiased sampling method and stained by periodic acid-Schiff (PAS) reagent. Mesangial matrix expansion was determined by assessing PAS-positive materials in the mesangial region excluding cellular elements. Percentage of PAS-positive area was analyzed using Image-Pro Plus (Media Cybernetics, Silver Spring, MD, United States) and Leica Q500MC image analysis software. Semi-quantitative analysis was performed with 30 glomeruli randomly selected fields for each subject (at least five mice in each group) and the evaluations were made by a blinded investigator.

### Reactive Oxygen Species (ROS) Measurement

Lipid peroxide concentration was determined by measuring the amount of malondialdehyde (MDA, SigmaAldrich) formed from thiobarbituric acid (TBA) during acid hydrolysis of lipid peroxides compound. Blood was harvested from heart with Heparin and centrifuged at 5000 rpm from 10 min at 4°C. Plasma was collected from supernatant. The reaction mixture contained 0.025 ml of sample, 0.025 ml of 8.1% sodium dodecyl sulfate (Sigma-Aldrich), 0.05 ml of 20% acetic acid solution and 0.075 ml of 0.67% thiobarbituric acid (Sigma-Aldrich). The mixture was then incubated at 95°C for 1 h. After cooling, the reaction mixture was centrifuged at 1, 10000 rpm for 10 min at 4°C. Absorbance of the supernatant layer was measured at 532 nm. Malonaldehyde *bis* (Sigma-Aldrich) was used to establish the standard curve. Lipid peroxide level was expressed in terms of nmol/L malondialdehyde per g protein.

### Plasma Cytokine Measurement

Inflammatory related molecules were measured in 25 μl triplicates by MILLIPLEX^®^ mouse cytokine/chemokine magnetic premixed bead panel immunoassay. This assay has a high sensitivity typically with a detection limit in the range from 0.01 to 0.48 ng/l.

### Arginase Activity Assay

Arginase activity was assayed by measuring urea produced from L-arginine as previously described (Romero et al., 2008). The plasma, the supernatant of hGECs lysate and kidney cortex homogenate, which is subjected to three freeze-thawcycles and centrifuged at 14,000 rpm for 10 min was collected. the fraction (25 μl) was heated with 25 μl MnCl_2_ (10 mM, 10 min, 56°C) to activate arginase. The mixture was then incubated with 50 μL of 0.5 M L-arginine (pH 9.7) at 37°C for 1 h. The reaction was stopped by adding acid; the solution was then heated at 100°C with 25 μl α-isonitroso-propiophenone (9% α-ISPF in ethanol) for 45 min. Samples were kept in the dark at room temperature for 10 min, and absorbance was then measured at 540 nm. Enzyme activity was normalized to the amount of protein assessed by Bradford protein assay.

### NO Production

Production of NO was measured using a Sievers 280i NO Analyzer. Media was collected from treated HGECs cultures and injected in glacial acetic acid containing sodium iodide in the reaction chamber. ${\text{NO}}_{2}^{-}$ is quantitatively reduced to NO under these conditions, which was quantified by a chemiluminescence detector after reaction with ozone. Addition of the NOS inhibitor L-NAME (100 μM, Cayman Chemical, CAS No. 51298-62-5) to control cultures prior to treatments reduced NO detection by over 95%.

### Quantitative Reverse Transcription-PCR (Q-PCR)

Total RNA from hGECs was isolated using TRIzol reagent (Invitrogen). Total RNA was reverse transcribed with M-MLV reverse transcriptase (Invitrogen) to generate cDNA. Gene expression was determined by quantitative PCR with SYBR Green Dye Gene Expression Assays for tumor necrosis factor (TNF-α), vascular cell adhension molecule-1 (VCAM-1), intracellular adhesion molecule 1 (ICALM-1) and Chemokine monocyte chemoattractant protein-1 (MCP-1) and heparanase or TaqMan Gene Expression Assays (for A2, Applied Biosystems), which was performed on a StepOne Plus thermocycler (Applied Biosystems). Primer sequences are presented in **Table 1**. The cycle threshold, determined as the initial increase in fluorescence above background, was determined for each sample. HPRT was used as internal control in the PCR reaction for normalization of assays.

**Table 1:** Primer sequences for expression of genes.

No.	Target	Forward (5′–3′)	Reverse (5′–3′)
1	ICAM-1	CAGTCCGCTGTGCTTTGAGA	CGGAAACGAATACACGGTGAT
2	VCAM-1	CTGGGAAGCTGGAACGAAGT	CAGGGGGCCACTGAATTGAA
3	MCP-1	GGCTCAGCCAGATGCAGTTAA	CCTACTCATTGGGATCATCTTGCT
4	TNF-a	GCTCTTACTGACTGGCATGAG	CGCAGCTCTAGGAGCATGTG
5	Heparanase	GGAGCAAACTCCGAGTGTATC	CAGAATTTGACCGTTCAGTTGG

### Western Blot Analysis

Mice kidney cortex tissues or hGECs were homogenized in lysis buffer containing protease inhibitors and phosphatase inhibitors and centrifuged at 14,000 *g* for 20 min at 4°C, supernatant collected and protein concentration determined. Proteins (25 μg) were resolved on a 10% SDS-polyacrylamide pre-cast gel and transferred to nitrocellulose membrane. The membranes were blocked in advance blocking agent (Amersham) and then incubated with primary antibody (anti-Arginase2: Santa Cruz Biotechnology, St. Louis, MO, United States, 1:500; anti-heparanase: Abcam, Cambridge, MA, United States, 1:1000) in 1% BSA in Tris-buffered saline/Tween 20 buffer overnight at 4°C. After washing, the membranes were incubated with sheep anti-mouse (Amersham, 1:4000) or donkey anti-rabbit (GE Healthcare, 1:4000) horseradish peroxidase-labeled secondary antibody and visualized using an enhanced chemiluminescence kit (Amersham, Piscataway, NJ, United States). The protein expression levels were normalized by actin or GAPDH.

### Immunofluorescence

Kidney sections (5 μm) were fixed in 4% paraffin and embedded in paraffin. Antigen retrieval and deparaffinization were performed using the antigen retrieval solution for 20 min at 95°C. All sections were blocked with 10% bovine serum albumin (BSA) and then incubated with monoclonal anti-mouse HS primary antibody (AMS Biotechnology, Milton Park, Abingdon, United Kingdom, 1:100), heparanase primary antibody (Abcam, Cambridge, MA, United States, 1:200) and arginase 2 primary antibody (Santa Cruz Biotechnology, Inc., Santa Cruz, CA, United States, 1:50) at 4°C overnight. The sections were washed in PBS and stained with rhodamine conjugated secondary antibodies for 60 min at room temperature. After washing in PBS, the sections were imaged by a Zeiss microscope (LSM-510, Carl Zeiss, Germany). Fluorescence intensity was quantified and analyzed by National Institutes of Health (NIH) Image software.

### Immunohistochemistry

Immunolocation of α-smooth muscle actin (α-SMA) was performed on paraffin-embedded section as previously described in detail (Xu et al., 2008) using following antibodies: α-SMA (Abcam, Cambridge, MA, United States, 1:500). α-SMA immunoexpression was assessed in 30 randomly selected glomeruli and/or cortical interstitial regions per section and quantitated using imagine analysis software (NIS-Elements, Ver. 4.6; Nikon Instruments, Melville, NY, United States). The data are expressed as percentage of area stained for α-SMA per selected area.

### Permeability Assay of hGECs Monolayers

Human glomerular endothelial cells monolayer permeability to high molecular mass proteins was assayed by using 2,000 kDa FITC-dextran (Sigma, St. Louis, MO, United States), base on the Tanswell Model (EMD Millipore) (Singh et al., 2011). hGECs were seeded on collagen-coated Transwells at a density of 1 × 105 cells per well in 250 μl of complete CSC medium. The inserts were placed into 24-well plates containing 500 μl of medium. Upon reaching 60% confluence, hGECs were exposed to HG as described above for 14 days with or without co-treatment with XSF containing serum and/or ABH. Transendothelial passage of dextran was determined after 14 days HG incubation. Medium was replaced by 150 μl of FITC-dextran in the insert for 3 h incubation. Then the insert was remove and 100 μl of medium was collected from the bottom chamber and transferred to a black 96-well plate. The fluorescent density of sample was analyzed on a Paradigm Microplate Fluorometer (Beckman-Coulter) at 485 nm excitation and 530 nm emission wavelengths.

### Statistical Analysis

All results were expressed as mean ± standard error (SE). Statistical analysis was performed using One-way ANOVA with a Turkey test by multiple comparison. A probability of *P* < 0.05 was considered to be statistically significant.

## Results

### Effect of XSF on Diabetes-Induced Renal Dysfunction

**FIGURE 1:**
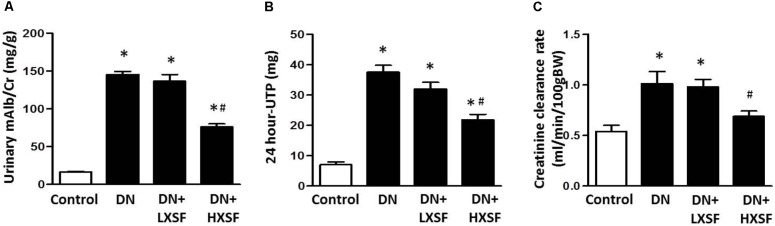
Xiao-Shen-Formula (XSF) ameliorated proteinuria and kidney impairments in DN mice. **(A)** The urinary microalbuminuria and creatinine ratio (mAlb/Cr), **(B)** 24 h-UTP and **(C)** creatinine clearance (CCr) were measured at 6 weeks after XSF treatment. Control: non-diabetic normal mice; DN: diabetic nephropathy; LXSF and HXSF: DN mice were treated with 1 or 3 g/kg/d XSF respectively. Data are presented as mean ± SE. ^∗^*P* < 0.05 vs. Control, ^#^*P* < 0.05 vs. DN, *n* = 10 mice/group.

The STZ-induced mice at the early stage showed elevated microalbuminuria and creatinine ratio (urinary mAlb/Cr), 24-h urinary total protein (24 h-UTP) and creatinine clearance (CCr), as compared with those of non-diabetic mice after 18 weeks diabetes. The 6 weeks-treatment of HXSF (3 g/kg/d) resulted in a significant suppression in all increases (**Figures 1A–C**). These data suggested that HXSF has therapeutic effect on renal hyper-filtration status in DN mice.

### Effect of XSF on Physiological and Biochemical Indexes

In our DN model, 12 weeks diabetes caused an increase in the ratio of kidney weight to body weight (KW/BW), the levels of blood glucose, including fast blood glucose and glycated hemoglobin, low density lipoprotein, angiotension II and aldosterone compared with control mice. HXSF treatment significantly decreased the ratio of KW/BW. However, other elevations were not altered by the treatment of either 1 or 3 g/kg/d XSF for 6 weeks. Furthermore, no XSF in low or high dosage can prevent the diabetes-induced reduction of BW (**Supplementary Table 1**). These results indicate that XSF treatment prevented diabetes induced renal hypotrophy, but did not exhibit any effect on the metabolic parameters and angiotensin–aldosterone system in DN mice.

### Effect of XSF on Diabetes-Damaged Glomerular Morphology

**FIGURE 2:**
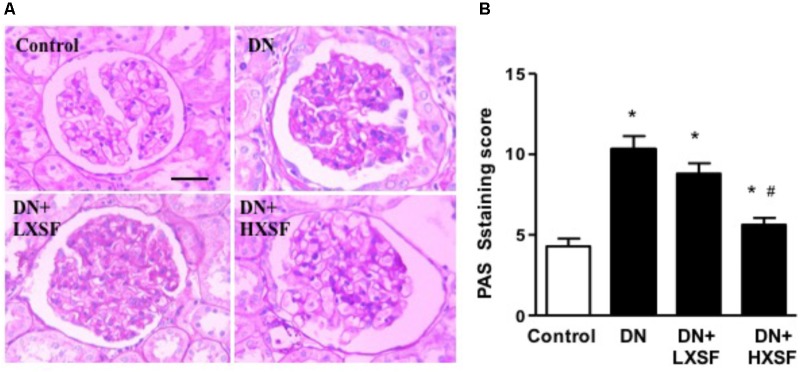
XSF prevented the damaged of glomerular morphology in DN mice. **(A)** Light microscopy of renal mesangial regions stained by PAS. Scale bar: 20 μm. **(B)** Quantitation of PAS staining were shown by PAS staining score. Data are presented as mean ± SE. ^∗^*P* < 0.05 vs. Control, ^#^*P* < 0.05 vs. DN, *n* = 6 mice/group.

To study whether XSF can prevent the glomerular impairment in diabetic condition, we assessed the extent of mesangial matrix expansion and the thickness of capillary basement membrane by using PAS staining. Diabetic mice demonstrated more glomeruli with higher level of mesangial matrix expansion and capillary basement membrane thickness, which was evaluated by PAS-stain score. Post-treatment of HXSF not LXSF largely reduce the extend (**Figure 2**). The results indicate that HXSF can treat the glomerulosclerosis in DN.

### Effect of XSF on Diabetes-Impaired Renal Microvascular Remodeling

**FIGURE 3:**
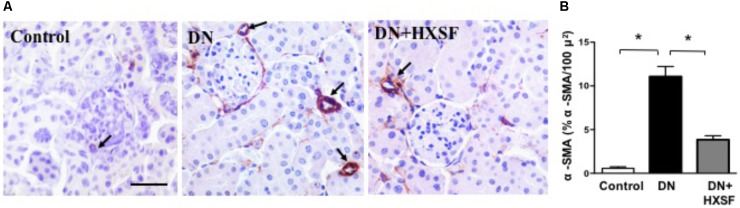
XSF prevented the impairment of microvascular remodeling renal cortical α-smooth muscle actin (α-SMA) density in DN mice. **(A)** α-SMA immunolocalization (brown staining). Scale bar: 50 μm. **(B)** Quantification of α-SMA density. Data are presented as mean ± SE. ^∗^*P* < 0.05, *n* = 5 mice/group.

To study the renal microvascular dysfunction, we examined the density of α-smooth muscle actin (α-SMA)-positive cells in cortical interstitium. Diabetes increased the density of α-SMA-positive cells by more than 20 fold after 18 weeks. HXSF treatment maintained the density in the similar level of control mice (**Figures 3A,B**), suggesting that HXSF prevented the progression of renal microvascular remodeling.

### Effect of XSF on Diabetes-Elevated Reactive Oxidative Species and Cytokines

**FIGURE 4:**
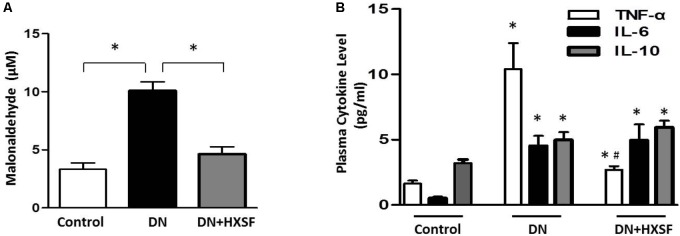
XSF blunted the elevation of reactive oxidative species and cytokines in DN mice. **(A)** Effect of XSF on plasma malonaldehyde (MDA). **(B)** Effect of XSF on plasma cytokines including proinflammatory cytokines tumor necrosis factor α (TNF-α) and IL-6 and anti-inflammatory IL-10. Data are presented as mean ± SE. ^∗^*P* < 0.05 vs. Control, ^#^*P* < 0.05 vs. DN, *n* = 5 mice/group.

As shown in **Figure 4A**, 18 weeks diabetic mice exhibited elevated oxidative stress as demonstrated by increased plasma lipid peroxides, which prevented by HXSF treatment, indicating the ameliorated level of ROS is at least partially involved in mechanisms of XSF treated DN. Further, there was an evidently increased proinflammatory cytokine level of TNF-α and IL-6 in diabetic mice (**Figure 4B**). Diabetes also raised the anti-inflammatory cytokine IL-10 levels, which was commonly induced in response to inflammation (**Figure 4B**). The post-treatment with HXSF only inhibited the elevation of TNF-α, but not altered the levels of IL-6 and IL-10, suggesting that XSF prevented inflammation in DN model through TNF-α involved signaling.

### Effect of XSF on the Expression of Glomerular Heparin Sulfate and Heparanase

**FIGURE 5:**
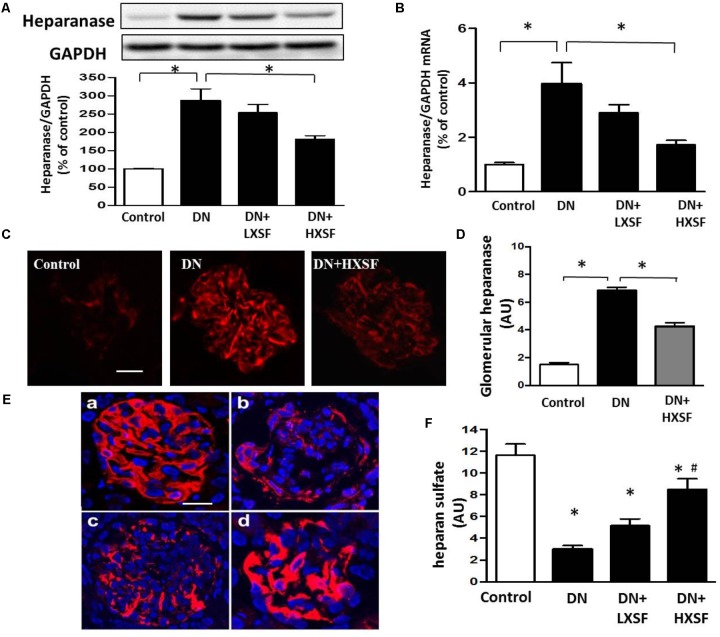
XSF inhibited cortical heparanase expression and restored the decreased glomerular heparan sulfate (HS) contents in DN mice. Effect of XSF on **(A,B)** protein and mRNA of heparanase; **(C)** the glomerular heparanase expression was determined by immunofluorescence staining. Scale bar, 20 μm. **(D)** The columns quantified the amount of immuno-reactive heparanase by NIH Image software. **(E)** The glomerular HS expression was determined by immunofluorescence staining. (a) Control; (b) DN; (c) LXSF; (d) HXSF. Scale bar, 20 μm. **(F)** The columns quantified the amount of immuno-reactive HS by NIH Image software. Data are presented as mean ± SE. ^∗^*P* < 0.05 vs. Control, ^#^*P* < 0.05 vs. DN, *n* = 5–10 mice/group.

In our DN model, the increases in protein and mRNA levels of heparanase especially in glomerular area were largely prevented by HXSF (**Figures 5A–D**). In associated with the increase of heparanase, the reduction of HS expression in glomerular region was recovered by HXSF (**Figures 5E,F**). These data suggest that HXSF prevented glomerular hyper-filtration by regulating heparanase expression in DN mice.

### Effect of XSF on the Level of Arginase Activity and Expression

**FIGURE 6:**
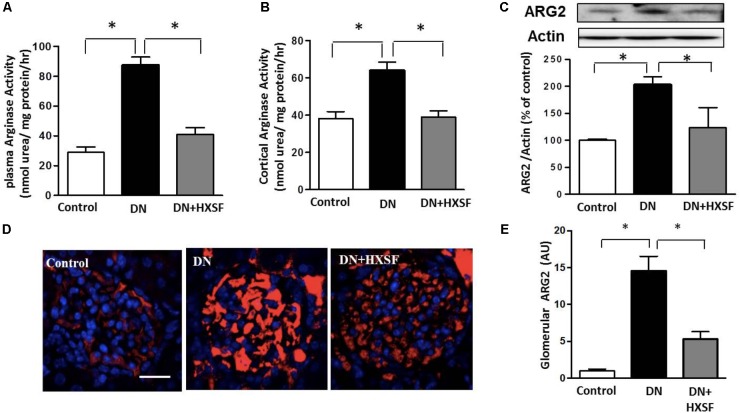
XSF attenuated plasma and cortical arginase activity and cortical arginase 2 (ARG2) expression. **(A)** Effect of XSF on plasma arginase activity, **(B)** cortical argianse activity, and **(C)** cortical ARG2 expression. **(D)** The glomerular ARG2 expression was determined by immunofluorescence staining. Scale bar: 20 μm. **(E)** The columns quantified the amount of immuno-reactive ARG2 by NIH Image software. Data are presented as mean ± SE. ^∗^*P* < 0.05, *n* = 5 mice/group.

To examine the effect of XSF on the arginase activation, arginase activity, and arginase 2 expression were detected. STZ-increased arginase activity in plasma and cortex were alleviated by treatment with HXSF (**Figures 6A,B**). This alleviation of arginase activity was correlated well with a reduction in arginase 2 expression in HSXF post-treated mice (**Figures 6C,D**). This result indicates that renal arginase 2 is one of primarily therapeutic targets of XSF in DN.

### Effect of XSF on Arginase-NO- Heparanase Pathway in High Glucose-Incubated hGECs

**FIGURE 7:**
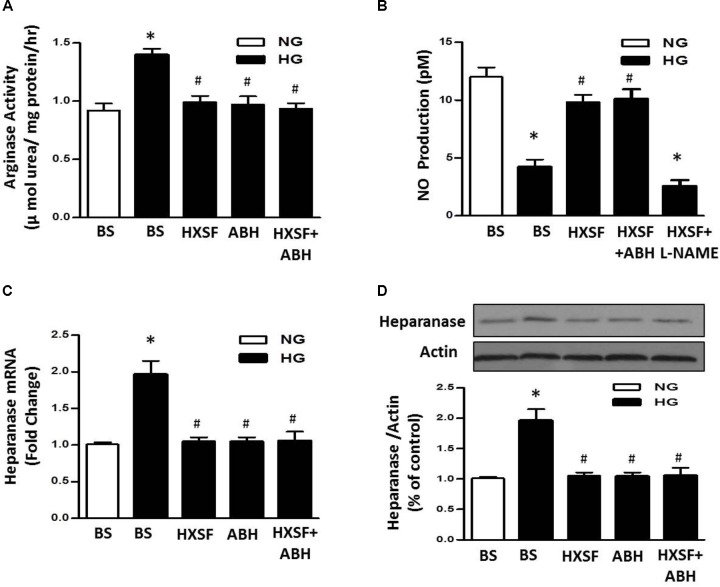
Effect of XSF on arginase-NO-heparanse pathway in hGECs. Effect of XSF (3 g/kg/d) containing serum (HSXF) on **(A)** arginase activity, **(B)** NO production, and **(C,D)** heparanse mRNA/protein in human glomerular endothelial cells (hGECs) with and without arginase inhibitor (ABH, 100 mM) under 24 h exposing to normal glucose (NG, 5 mM) and high glucose (HG, 25 mM). BS: basal serum. Data are presented as mean ± SE. ^∗^*P* < 0.05 vs. Control, ^#^*P* < 0.05 vs. DN, *n* = 5 experiments.

We further investigated whether the level of heparanase protein can be regulated by arginase activation in high glucose (HG) treated hGECs. The enhancement of arginase activity is completely inhibited by post-treated with ABH (100 μM) or mouse serum containing HXSF (MS-HXSF, 3 g/kg/d). The hGECs presented in HG with both MS-HXSF+ABH did not cause further reduction of arginase activity comparing with ABH treatment alone (**Figure 7A**), indicating XSF has similar effect with arginase inhibitor on controlling arginase activity in HG medium.

Furthermore, Post-treatment with MS-HXSF prevented the elevation of heparanase protein/mRNA expression and the reduction of NO production. There was similar effect by present of ABH or MS-HXSF+ABH compared with MS-HXSF alone (**Figures 7B–D**). This preventive effect in NO reduction by MS-XSF was eliminated by NOS inhibitor, L-NAME (100 μM) (**Figure 7B**).

### Effect of XSF on Inflammatory Reaction and the Permeability of hGECs Monolayers Exposed to High Glucose

**FIGURE 8:**
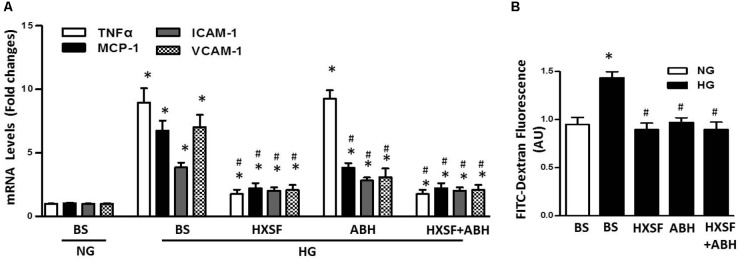
Effect of HXSF containing serum on the permeability of hGECs and mRNA of cytokine, chemokine, and adhesion molecules in hGECs. **(A)** The levels of proinflammatory cytokines TNF-α, the chemokine monocyte chemoattractant protein-1 (MCP-1), and the adhesion molecules vascular cell adhesion molecule-1 (VCAM-1) and intercellular adhesion molecule 1 (ICALM-1). **(B)** Transendothelial passage of FITC-dextran was used to determine permeability of hGECs monolayer, seeded onto collagen-coated transwells. Fluorescent density of samples was analyzed on a paradigm microplate fluorometer. BS, basal serum. Data are presented as mean ± SE. ^∗^*P* < 0.05 vs. Control, ^#^*P* < 0.05 vs. DN, *n* = 5 experiments.

We further found that HG significantly increased levels of mRNA for TNF-a, intercellular adhesion molecule 1 (ICAM-1), vascular cell adhesion protein 1 (VCAM-1) and monocyte chemoattractant protein-1 (MCP-1) compared with normal glucose, which were prevented by MS-HXSF (**Figure 8A**). However, ABH post-treatment blunted all the elevation, except the rise of TNF-α. The results indicated that XSF intervened other anti-inflammatory mechanism in diabetes-induced glomerular endothelium inflammation as well besides the arginase-NO pathway. Post-treated of ABH, or MS-HXSF conferred a significant protection from HG-induced hGECs hyper-permeability (**Figure 8B**). There is no synergistic effect in present both MS-HXSF+ABH. These results suggest that XSF maintains the integrity of glomerular endothelial barrier at least in part by reducing arginase activity.

## Discussion

The major findings as follows: first, post-treatment of HXSF prevented STZ-induced renal injury, but did not alter the metabolic parameters and angiotensin–aldosterone system in DN mice (**Figure 1** and **Table 1**). Second, post-treatment of HXSF attenuated DN-caused glomerulosclerosis and renal microvascular remodeling, associated with decreasing the higher ratio of KW/BW (**Figures 2, 3** and **Table 1**). Third, post-treatment of HXSF ameliorated the diabetes-elevated ROS, inflammatory cytokines and glomerular hyper-filtration, companied with lower levels of arginase activity, arginase 2 and heparanase expression and levels of HS expression in glomerular region (**Figures 4–6**). Moreover, we revealed that XSF protected HG-induced hyper-permeability and inflammation in hGECs by inhibiting arginase–heparanase pathway (**Figures 7, 8**).

Consistent of our previous studies using XSF in type 2 DN patients (Teng and An, 2016; An et al., 2017), we found that post-treatment of XSF prevented STZ-induced renal injury in mice. The prevention of glomerular endothelium dysfunction is key target for the intervention in the progression of DN (Gnudi et al., 2016). Current initial treatments of DN include controlling the normal levels of blood glucose and blood pressure (UK Prospective Diabetes Study [UKPDS] Group, 1998a,b; Fowler, 2008). However, blockade of rein angiotensin system did not reduce the microalbuminuria and prevent the renal injury in the end-stage of type 2 DN (Vejakama et al., 2012). Our present study found that post-treatment of HXSF prevented STZ-induced renal injury in mice without affecting the metabolic syndrome and angiotensin–aldosterone system (**Figure 1** and **Table 1**).

Previous studies reported that an increased glomerular heparanase expression can induce and enhance proteinuria by degrading HS in GBM (van den Hoven et al., 2007). And eNOS-NO pathway as a new mechanism was involved in the regulation of heparanase expression (Maxhimer et al., 2005; Shafat et al., 2006; van den Hoven et al., 2009; Masola et al., 2011; Garsen et al., 2016b). Our previous studies found that inhibition of arginase activity in mouse aortic endothelial cells effectively prevented vascular dysfunction and maintained levels of NO (Bhatta et al., 2017). Systemic inhibition of arginase activity or genetic knockout arginase 2, effectively restored the reduction of renal medulla blood glow and renal dysfunction (Morris et al., 2011). All these studies suggesting that intervening arginase activation can prevent renal damage through controlling NO-heparanase pathway. We for the first time demonstrate that arginase activation is involved in the regulation of heparanase in DN.

Accumulated researches revealed that the induction of inflammation and oxidative stress by the metabolism of hyperglycemia and dyslipidemia played significant roles in developing vascular complications (Banba et al., 2000; Sassy-Prigent et al., 2000; Gaede et al., 2001; Okada et al., 2003; Chow et al., 2004; Mima, 2013). Consistent with above studies, we found that post-treated XSF largely reduced the level of systemic ROS and inflammatory cytokines, TNF-α. Post-treatment of MS-XSF prevented the inflammation of hGECs by decreasing the levels of ICAM1, VCAM1, MCP-1, and TNF-α in HG maintained medium. Heparanase-driven molecular events have been reported to promoting TNF-α and renal injury, including TNF-α signaling (Goldberg et al., 2014), which support our current results. However, our previous works found that inhibition of arginase activity by ABH and knockout arginase 1 in endothelial cells effectively alleviated the enhancement of ROS and vascular inflammation in type 2 diabetes mice model, but TNF-α was not involved (Yao et al., 2017). Considering the characteristics of traditional Chinese medicine are multi-components and multi-targets (Han et al., 2017), arginase signaling may be only one of them. Taken all results together, it suggests that arginase is involved in XSF preventing inflammation in diabetic renal injury.

As an inner layer of glomerular filtration barrier, GECs impairment has been proved to be correlated with the urinary albumin creatinine ratio (Toyoda et al., 2007; Weil et al., 2012), which happened before podocytes and GBM damage in rodent and human diabetes (Jeansson et al., 2006; Broekhuizen et al., 2010). These results indicate that besides the podocytes, GECs is also a significant target in treatment of DN. Healthy vascular endothelium is covered by the endothelial glycocalyx, and the structural integrity of a glomerular endothelial glycocalyx is crucial to regulate the GECs permeability and inflammation (Meuwese et al., 2010; Dane et al., 2013; LWMM Rops et al., 2014). Increased activity of heparanase reduces the dimensions of the glomerular endothelial glycocalyx by degradation of HS, which has long been recognized in DN (van den Hoven et al., 2006; Gil et al., 2012). Loss of endothelial cell function is highly associated with kidney dysfunction in many diabetic patients who are characterized by albuminuria/proteinuria (Ndisang, 2018). And eNOS prevents the development of proteinuria through heparanase regulation in diabetic mouse model (Garsen et al., 2016b). All these researches suggests that NO-heparanase signaling is a crucial target for the treatment of GECs barrier damage in diabetic neuropathy. Our results showed that XSF treatment prevented HG-elevated heparanase expression, arginase activity and impaired NO synthesis, which is correlated with the reduction of glomerular endothelial permeability. Meanwhile, co-treatment of XSF with ABH did not cause any difference compare with the ABH or XSF alone, indicating that arginase in GECs is one of XSF therapeutic targets to restored HG-elevated glomerular endothelial permeability. All results suggest that glomerular endothelial arginase is highly involved in DN-induced glomerular dysfunction.

**FIGURE 9:**
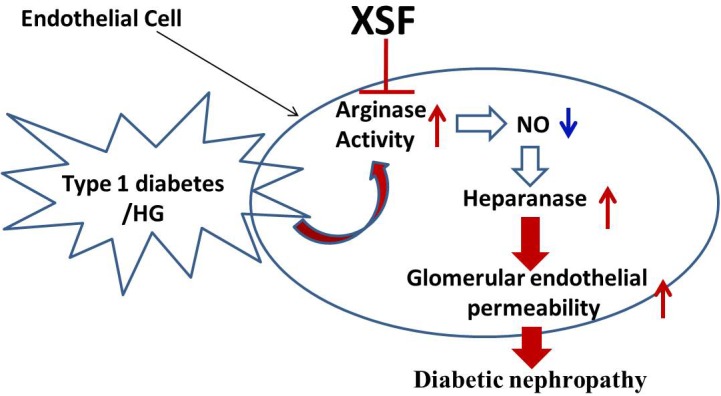
Schematic diagram representing XSF on amelioration of heparanase expression and improvement of glomerular hyperpermeability by inhibtion of arginase activation.

In conclusion, this study demonstrated that XSF ameliorated STZ-induced renal failure through inhibiting arginase activity and heparanase protein expression in GECs contributes to the therapeutic effect of XSF (**Figure 9**).

## Ethics Statement

This study was carried out in accordance with the recommendations of Guide for the Care and Use of Laboratory Animals published by the US National Institutes of Health (NIH Publication, 8th Edn, 2011). The experimental protocol was approved by the Committee on Ethics of Guangzhou University of Chinese Medicine.

## Author Contributions

LY, MZ, and YC conceived and designed the protocol. LY, XA, TL, and SZ performed the experiments. LY, XA, and TL analyzed the data. LY wrote the paper. All the authors reviewed and approved the submitted version of the paper.

## Conflict of Interest Statement

The authors declare that the research was conducted in the absence of any commercial or financial relationships that could be construed as a potential conflict of interest.
