# Supplementary material for: Xiao-Shen-Formula, a Traditional Chinese Medicine, Improves Glomerular Hyper-Filtration in Diabetic Nephropathy via Inhibiting Arginase Activation and Heparanase Expression
Source: Front Physiol. 2018 Sep 26;9:1195. doi: 10.3389/fphys.2018.01195 (PMC6169603; doi:10.3389/fphys.2018.01195)
Supplement: Supplementary file 1 [file Table_1.doc]

Table 1. The effect of XSF treatment on physiological and biochemical indexes in diabetic nephropathy mice (n=10).

| Index | Control | DN | LHPS | HHPS |
| --- | --- | --- | --- | --- |
| BW (g) | 26.6±2.4 | 23.5±2.1^＊^ | 24.3±2.5^＊^ | 24.6±2.2^＊^ |
| KW / BW (mg/g) | 11.7±0.7 | 13.9±0.6^＊^ | 13.3±0.7^＊^ | 11.9±0.5^＃^ |
| FBG (mmol/L) | 5.4±0.6 | 16.2±2.2^＊^ | 16.4±2.5^＊^ | 15.8±2.4^＊^ |
| HbA1c (%) | 5.5±0.7 | 10.4±1.6^＊^ | 10.2±1.5^＊^ | 9.8±1.3^＊^ |
| LDL (mmol/L) | 2.4±0.4 | 3.0±0.5^＊^ | 2.8±0.6^＊^ | 2.9±0.6^＊^ |
| Ang II (pg/ml) | 63.4±7.2 | 72.5±8.6^＊^ | 69.3±8.8^＊^ | 67.6±9.2^＊^ |
| Aldosterone (pg/ml) | 113.2±13.4 | 146.8±15.3^＊^ | 138.2±14.2^＊^ | 136.7±14.6^＊^ |

BW, Body Weight; KW, Kidney Weight; FBG, fasting blood glucose; HbA1c, glycated hemoglobin; LDL, Low-density lipoprotein; Ang II, angiotensin II; Control: Normal mice; DN: diabetic nephropathy mice were treated with vehicle solution; LXSF and HXSF: DN mice were treated with 1 g/kg/d or 3g/kg/d XSF for 6 weeks respectively. Data are presented as mean ± SE. P<0.05 is statistically significant. ^＊^ indicates significant vs. Control.
